# Phage Display Reveals VLRB-Mediated Recognition of Minimal Tumor Glycan Antigen Sialyl-Tn

**DOI:** 10.3390/cimb47100802

**Published:** 2025-09-26

**Authors:** Mark Rickard N. Angelia, Abigail Joy D. Rodelas-Angelia, Youngrim Kim, Cheolung Yang, Hyeok Jang, Seungpyo Jeong, Jihyun Mun, Kim D. Thompson, Taesung Jung

**Affiliations:** 1 Laboratory of Aquatic Animal Diseases, Institute of Animal Medicine, College of Veterinary Medicine, Gyeongsang National University, 501, Jinju-Daero, Jinju-si 52828, Republic of Korea; 2 Institute of Chemistry, College of Arts and Sciences, University of the Philippines Los Baños, College, Laguna 4031, Philippines; 3 Moredun Research Institute, Pentlands Science Park, Bush Loan, Midlothian EH26 0PZ, UK; 4 Earwynbio Co., Ltd., 206 Sungjangjiwon-dong, 991 Worasan-ro, Munsan, Jinju-si 52839, Republic of Korea

**Keywords:** variable lymphocyte receptor, phage display, sialyl Tn, TACA, glycan-binding

## Abstract

Sialyl-Tn (sTn) is a tumor-associated carbohydrate antigen (TACA) abundantly expressed by various types of carcinomas. While conventional antibody-based platforms have traditionally been used for the detection and targeting of sTn, alternative binding scaffolds may offer distinct advantages. Variable lymphocyte receptor B (VLRB), the immunoglobulin-like molecule of jawless vertebrates, offers a promising alternative for glycan recognition. In this study, a phage-displayed VLRB library was utilized to identify sTn-specific binders. Two candidates, designated as ccombodies A8 and B11, were isolated after four rounds of biopanning. Both were expressed and purified using Ni-affinity and FPLC, yielding proteins with apparent molecular weights of ~27 kDa in SDS-PAGE. Sequence analysis revealed a preference for glycan-binding residues in randomized hypervariable regions, with A8 exhibiting an increased aliphatic content. ELISA confirmed selective binding to sTn and other *O*-glycans containing the core α-GalNAc, with EC_50_ values of 18.2 and 14.2 nM for A8 and B11, respectively. *Vicia villosa* lectin inhibited ccombody binding to sTn, indicating shared epitope recognition. Additionally, both ccombodies bound to sTn-positive glycoproteins and carcinoma cell lines HeLa and LS174T. These findings demonstrate that phage display of VLRBs enables the identification of high-affinity, glycan-specific binders, offering a compelling alternative to immunoglobulin-based platforms for future diagnostic and therapeutic applications targeting tumor-associated glycans.

## 1. Introduction

Aberrant glycosylation is a hallmark of cancer, frequently observed in glycan-bearing molecules such as glycoproteins, glycolipids, and proteoglycans. These changes are primarily driven by abnormal or ectopic expression of glycosyltransferases and glycosidases, as well as dysregulation of sugar nucleotide donor supply [1]. Such alterations give rise to tumor-associated carbohydrate antigens (TACAs), which are implicated in tumor progression and immune evasion. Common glycosylation changes include hypersialylation, *O*-glycan truncation, increased *N*-glycan branching, and altered fucosylation patterns [2,3]. Among the most extensively studied TACAs are the glycoprotein-linked Thomsen-nouveau (Tn), sialyl-Tn (sTn), and Thomsen–Friedenreich (TF); blood group antigens Lewis^X^ and Lewis^Y^, including their sialylated forms; GD2, GD3, GM2, GM3, and fucosyl-GM1 gangliosides; and globo-series such as GB3, GB4, GB5, and Globo-H [4].

Sialyl-Tn antigen, also known as CD175s, is a disaccharide consisting of sialic acid (Neu5Ac) α2-6 linked to *N*-acetyl-galactosamine (GalNAc), which in turn is linked to either serine or threonine residue of a protein via an *O*-glycosidic bond. Addition of sialic acid to GalNAc is catalyzed by ST6GalNAc1, a sialyltransferase enzyme. This sialylation blocks further extension and biosynthesis of more complex *O*-glycan core structures, resulting in the formation of short, truncated *O*-glycans [5]. While sTn expression is limited in normal cells, it is abundantly expressed in many types of cancer cells [6,7]. Altered expression or mutation of key enzymes involved in *O*-glycan biosynthesis, such as core1-specific-molecular-chaperone (COSMC), core1 β3-galactosyltransferase (T synthase), and ST6GalNAc1, leads to the overexpression of sTn in cancer and other pathological conditions [8]. Given its characteristic tumor-restricted overexpression, the reliable detection of sTn has become a focus for the development of antibody-based diagnostic and therapeutic tools [9,10,11,12]. However, several challenges remain to be addressed for antibody-based technologies targeting sTn, including cross-reactivity, epitope masking or shielding, low immunogenicity, conformational flexibility, and suboptimal epitope presentation [13,14].

Variable lymphocyte receptors (VLRs), found in lampreys and hagfish (agnathans), confer immune protection analogous to that of conventional immunoglobulin-based antibodies in jawed vertebrates (gnathostomes). Three distinct classes of receptors have been identified, namely VLR A, B, and C, each associated with specialized immune functions. VLRA and VLRC are expressed by VLRA^+^ and VLRC^+^ lymphocytes, which are considered functional counterparts to αβ^+^ and γδ^+^ T lymphocytes, respectively. In contrast, VLRB is expressed by VLRB^+^ lymphocytes, which serve a similar role to B cells by secreting soluble antigen-specific receptors [15]. In terms of structure, VLRs adopt a crescent-shaped topology due to several combining leucine-rich repeat (LRR) modules, reminiscent of Toll-like receptors (TLRs). The antigen-binding surface is located on the concave face formed by continuous β-sheets generated from adjoining LRR modules. Mature VLRs are composed of the following segments in sequence: a signal peptide (SP), an N-terminal LRR cap (LRRNT), an initial LRR (LRR1), one to seven variable LRRs (LRRVs), a terminal variable LRRV (LRRVe), a connecting peptide (CP), a C-terminal LRR cap, and an invariant stalk region [16,17]. Among the VLR classes, VLRB is the best characterized and has demonstrated remarkable specificity and affinity to various types of antigens. VLRB has been applied across various fields, including pathogen detection [18], biomarker discovery [19], whole cell antigen screening [20], therapeutics [21], aquaculture [22], and agriculture [23]. Moreover, the unique structural architecture of VLRBs enables them to recognize and bind carbohydrate epitopes, an increasingly valuable characteristic in glycobiology and cancer research [15,24].

In this study, we engineered a novel synthetic scaffold based on consensus-designed hagfish VLRB molecules. Using phage display technology, we screened this scaffold to isolate receptors with specificity for the sTn antigen. For clarity and consistency, we refer to these engineered VLRBs as “ccombodies” throughout this work.

## 2. Materials and Methods

### 2.1. Phage Display Library Screening for STn-Binding Ccombodies

#### 2.1.1. Phage-Displayed Ccombody Library Construction

The phage display library was constructed according to the methods of Sirimanapong et al. [25]. Briefly, synthetic hagfish ccombody sequences, designed by merging natural hagfish ccombody consensus LRR (leucine-rich repeat) modules, were fused with the N-terminus of M13 pIII minor coat protein. The constructed ccombodies contain eight possible randomization sites encoded within seven LRR and one CP segments, corresponding to the hypervariable regions (Table 1). For this screening, the sequences of segments 1–4 were fixed, while 5–8 were randomized, yielding a total diversity of approximately 6.6 × 10^20^.

#### 2.1.2. Antigen Coating and Blocking

A 100 µL (containing 1 mg) of streptavidin-coated magnetic beads (Dynabeads, Invitrogen, Waltham, MA, USA) was prewashed according to the manufacturer’s specifications prior to antigen conjugation. Next, 200 pmol of biotinylated-sTn antigen (Sussex Research Laboratories, Inc., Ottawa, ON, Canada) was subsequently conjugated to the magnetic beads by incubation for 1 h with gentle mixing on a rotary mixer. Excess unbound antigen was removed by washing the beads three times with 1× phosphate-buffered saline (PBS; 135 mM NaCl, 2.7 mM KCl, pH 7.4) containing 0.05% Tween 20 (PBST), then once with PBS. Prior to biopanning, the beads were blocked with 1% bovine serum albumin (BSA) in 1× PBST (blocking buffer 1) by gentle rotary mixing for 1 h, then washed twice with PBST.

#### 2.1.3. Bead-Based Biopanning

The following steps describe one round of bead-based biopanning [26]. Briefly, negative preselection was initially performed, whereby the phage library, consisting of 10^12^ plaque-forming units (pfu) in 1 mL blocking buffer 1, was added onto “uncharged” beads (no antigen), followed by gentle rotation for 1 h at room temperature. Unbound phages were removed from the mixture and then immediately transferred onto antigen-loaded beads, followed by gentle rotation for another hour. The beads were washed four times with PBST, then once with PBS to remove nonbinding phages. Bound phages were then eluted by the addition of 1 mL 0.25 M glycine, pH 2.2, after standing for 10 min. The eluate was then collected and transferred into a 50 mL conical tube containing 200 µL of 1 M Tris, pH 9.0, for neutralization. Then, 9 mL of early-log phase *E. coli* TG1 was added for subsequent infection by the pooled phage particles. The mixture was incubated for 1 h at 37 °C and 200 rpm, after which phage-infected cells were pelleted by centrifugation (Hanil Science Co., Ltd., Daejeon, Republic of Korea) and cultured on a 2× YT plate (1% glucose, 100 µg/mL ampicillin) at 30 °C overnight. The following day, cells were scraped and resuspended in 5 mL of 2× YT. An aliquot was grown in 50 mL of 2× YT broth (1% glucose, 100 µg/mL ampicillin) at 37 °C and 200 rpm, until cells were in the early- to mid-log phase, corresponding to an OD_600_ of 0.5–0.7. Next, 100 µL of M13KO7 (Antibody Design Laboratories, San Diego, CA, USA) helper phage was added and allowed to infect cells via incubation for 1 h at 37 °C and 200 rpm. Infected cells were collected via centrifugation, then inoculated in 200 mL of 2× YT broth (0.5 mM IPTG, 100 µg/mL ampicillin, and 50 µg/mL kanamycin) and grown overnight at 30 °C and 200 rpm. Subsequently, the culture supernatant was collected via centrifugation for 20 min at 4 °C and 8000 rpm, then added with 50 mL of 20% (*w*/*v*) polyethylene glycol solution (PEG 8000, 2.5 M NaCl) to precipitate the phage particles. Finally, the phage particles were recovered by centrifugation for 1 h at 4 °C and 8000 rpm. The phage titer was determined from absorbance measurement at 260 nm, then adjusted to 10^12^ pfu for the next round of biopanning. Four rounds of biopanning were performed to enrich the phage pool with sTn binders.

#### 2.1.4. Colony Isolation and Propagation

Colonies obtained from the last round of biopanning were picked and grown in a deep-well plate (96-well) containing 100 µL of 2× YT broth (100 µg/mL ampicillin), for 3 h at 37 °C and 200 rpm. Next, helper phage was added to infect the cells after incubation for 1 h at 37 °C and 200 rpm. Then, infected cells were allowed to multiply in 200 µL of 2× YT broth (100 µg/mL ampicillin and 50 µg/mL kanamycin) for at least 16 h at 30 °C and 200 rpm. Culture supernatants, which contain the ccombody-displaying phages, were separated by centrifugation at 3500 rpm for 10 min, then used in phage ELISA as described below.

#### 2.1.5. Phage ELISA Screening

A 100 µL of biotinylated-sTn antigen (250 pmol/well) in PBS buffer was added into a 96-well streptavidin-coated high-binding capacity ELISA plate (ThermoFisher Scientific, Waltham, MA, USA) and allowed to bind overnight at 4 °C. BSA, coated onto a high-binding 96-well ELISA plate (Corning, Corning, NY, USA), served as a negative control. Unbound antigens were removed by washing the plates three times with 1× TBST (10 mM Tris-HCl, 150 mM NaCl, and 0.5% Tween 20). The BSA plate was further blocked with 5% skimmed milk in 1× TBST (blocking buffer 2) for 1 h at room temperature, then washed twice with 1× TBST. Next, 100 µL of culture supernatants was added into the plates, incubated for at least 1 h 30 min, then washed three times with 1× TBST. Antigen-specific ccombody-displaying phages were detected using HRP-conjugated mouse anti-M13 antibody (Sino Biological, Beijing, China) following incubation for 1 h, then washing four times with 1× TBST. Subsequent colorimetric reaction was induced by the addition of 100 µL developing buffer containing 42 mM 3,3′,5,5′-tetramethylbenzidine (TMB) and 1% H_2_O_2_. Reaction was terminated by the addition of 50 µL of 2 M H_2_SO_4_. Absorbance was then read at 450 nm using an *x*Mark microplate reader (Bio-Rad Laboratories, Inc., Hercules, CA, USA). Phage clones that exhibited strong and selective binding with the antigen were further sequenced and subcultured for plasmid purification and cloning into the expression vector.

#### 2.1.6. Cloning and Expression of STn-Specific Ccombodies

Plasmid DNA carrying the gene for sTn-specific ccombodies was purified from *E. coli* TG1 cells using Hybrid-Q Plasmid Rapidprep(200) kit (GeneAll, Seoul, Republic of Korea), according to the manufacturer’s specifications. Next, ccombody gene amplification and shuttle vector (developed in-house) linearization were performed via PCR using the appropriate primer pairs. Circular polymerase extension cloning (CPEC) was then employed to join the gene of interest with the vector. Finally, electrocompetent *E. coli* BL21(DE3) was used for plasmid transformation via electroporation using a MicroPulser Electroporator (Bio-Rad Laboratories, Inc., Hercules, CA, USA). Following IPTG induction, the transformants were grown overnight in 200 mL of LB media at 20 °C and 200 rpm to allow expression of recombinant sTn-specific ccombodies. The shuttle vector incorporates an N-terminal 6×His-tag into the ccombody sequence to enable affinity purification using nickel-charged magnetic beads.

#### 2.1.7. Purification of Recombinant STn-Binding Ccombodies

Affinity purification, using PureCube INDIGO Ni-MagBeads (Cube Biotech, Monheim, Germany), was employed to isolate the His-tagged sTn-binding ccombodies from the rest of the bacterial proteins. The eluate obtained from the previous step was then loaded into a fast protein liquid chromatography (FPLC) system using a Superdex^TM^ 75 Increase 10/300 GL column (ÄKTA go, Cytiva, Marlborough, MA, USA). Fractions were monitored by SDS-PAGE using 10% polyacrylamide gels. Fractions containing the sTn-binding ccombodies were pooled and subjected to further characterization.

### 2.2. Characterization of STn-Binding Ccombodies

#### 2.2.1. Assessment of Ccombody Specificity via ELISA

Ccombody specificity was evaluated using ELISA as described earlier, with minor modifications. Various biotinylated glycans and glycoproteins were added to streptavidin-coated and regular high-binding ELISA plates, respectively, followed by an overnight incubation at 4 °C. After blocking with 5% skimmed milk (in blocking buffer 2), 100 µL of the purified sTn-binding ccombodies was added and allowed to bind for 1 h at room temperature. After incubation, plates were washed three times with 1× TBST to remove the unbound ccombodies. Next, 100 µL of mouse anti-ccombody antibody (5C12; developed in our lab) was added to detect the bound ccombodies. Subsequently, the plates were washed and incubated with HRP-conjugated goat anti-mouse antibody (ThermoFisher Scientific, Waltham, MA, USA), followed by color development using TMB substrate. To stop the reaction, 50 µL of 2 M H_2_SO_4_ was added, and the absorbance was measured at 450 nm.

#### 2.2.2. EC_50_ Determination of Ccombody-sTn Binding

EC_50_ (half-maximal binding) was determined using an ELISA as described earlier, with minor modifications. Biotinylated-sTn antigen was coated at 250 pmol/well, followed by the addition of serial two-fold dilutions (ranging from 0.20 to 9.54 × 10^−8^ mg/mL) of ccombodies. The background corrected absorbance values (450 nm), as well as the ccombody concentration (mg/mL), were fitted into the four-parameter logistic (4PL) equation [27] to determine the EC_50_ value. Values were expressed in nanomolar units and reported as the mean of two measurements.

#### 2.2.3. Immunofluorescence

For immunofluorescence studies, the following cell lines were used: HeLa (kindly provided by Dr. Sang Soo Kang of the College of Medicine, Gyeongsang National University, Jinju, Republic of Korea), LS174T (Korean Cell Line Bank, Seoul, Republic of Korea), and HEK293F (ThermoFisher Scientific, Waltham, MA, USA). The cells were grown to 70–80% confluence in Nunc MicroWell 96-well Microplates (ThermoFisher Scientific, Waltham, MA, USA) at 37 °C with 5% CO_2_ in a humidified incubator. After media removal and washing, cells were fixed with 4% paraformaldehyde for 15 min at room temperature. Thereafter, the cells were processed in the following sequence, with washings between steps: (a) blocking with 5% skimmed milk in PBST, (b) incubation with sTn-binding or unrelated ccombody (negative control), (c) incubation with 5C12, and (d) incubation with FITC-conjugated goat anti-mouse antibody (Invitrogen). The cell nuclei were also visualized with Hoechst 33342 (ThermoFisher Scientific, Waltham, MA, USA). Fluorescent signals were viewed and recorded on a Nikon Eclipse T*i* inverted microscope (Nikon Instruments Inc., Melville, NY, USA). Instrument exposure and gain were kept constant for all measurements. *Vicia villosa* lectin (VVL) (Vector Laboratories, Newark, CA, USA) was included as a positive control and separately detected using streptavidin-FITC (Invitrogen, Waltham, MA, USA).

#### 2.2.4. Lectin Competition Assay

Ccombody binding was analyzed using the ELISA as presented earlier, with minor modifications. Two-fold serial dilutions (5 to 0 µg/mL) of VVL were preincubated with a sub-saturating concentration of sTn-binding ccombodies (previously determined from titration) for 1 h at room temperature. Following the blocking step, 100 µL of the lectin-ccombody mixture was added onto the biotinylated-Tn and sTn coated ELISA plates, then incubated for 1 h at room temperature. Bound ccombodies were detected as described previously. Ccombody binding was expressed as percent binding, and percent inhibition was computed using the formulas below:

$$\%\mathrm{Binding}\;=\;\frac{\left({OD}_{sample}-{OD}_{blank}\right)}{({OD}_{Ccombody\;only}-{OD}_{blank)}}\;\times 100$$

%Inhibition = 100 − %Binding

## 3. Results

### 3.1. Phage Display Library Screening

**Figure 1:**
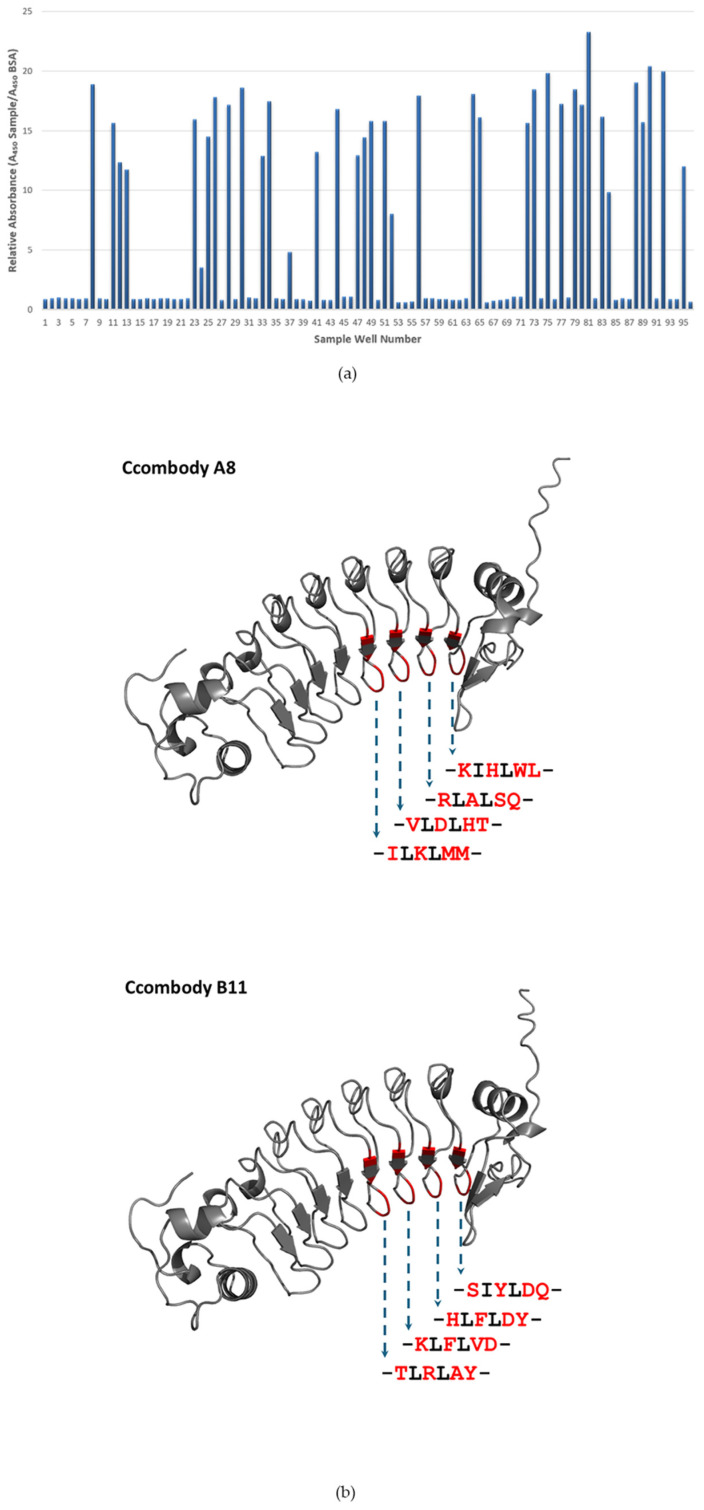
(**a**) Monoclonal ELISA of phage-displayed ccombodies against biotinylated sTn antigen. Signals are expressed in relative absorbance versus BSA. (**b**) Predicted 3D-structures of ccombodies A8 and B11 [29] are shown, with the randomized amino acids highlighted in red. The actual sequences of the peptide segments are presented below each structure.

We employed a large repertoire phage-displayed ccombody library to isolate binders specific to sTn. Bead-based biopanning was chosen for this purpose because it offers potentially greater efficiency in isolating high-affinity phages [28]. After four rounds of biopanning, the phage population was expected to be enriched with ccombodies specific to the glycan antigen. The initial negative preselection step also helped eliminate binders that targeted the bead scaffold and other matrix components, thus minimizing non-specific binding artifacts. M13 ELISA (Figure 1a) yielded several candidates with strong binding signals, greater than 10-fold relative to the negative control (BSA). To ensure the selection of potent binders, clones displaying signal intensities exceeding 15-fold higher relative to the control were chosen for subsequent experiments. Nucleotide sequencing, however, revealed only two unique sTn-binding candidates, designated as ccombodies A8 and B11. The amino acid residues at the randomized positions of these ccombodies showed an apparent preference for polar-uncharged, charged, and aromatic side chains, which are key contributors for glycan binding (Figure 1b). Interestingly, ccombody A8 showed a greater preference than B11 for non-polar aliphatic residues in the randomized positions (Table 2). This observation may challenge the conventional view that aliphatic residues are generally unfavored in the binding sites of glycan-binding proteins due to their hydrophobic nature. However, collectively, residues that favor hydrogen bonds and polar interactions remained predominant over those mediating hydrophobic contacts.

### 3.2. Ccombody Purification

**Figure 2:**
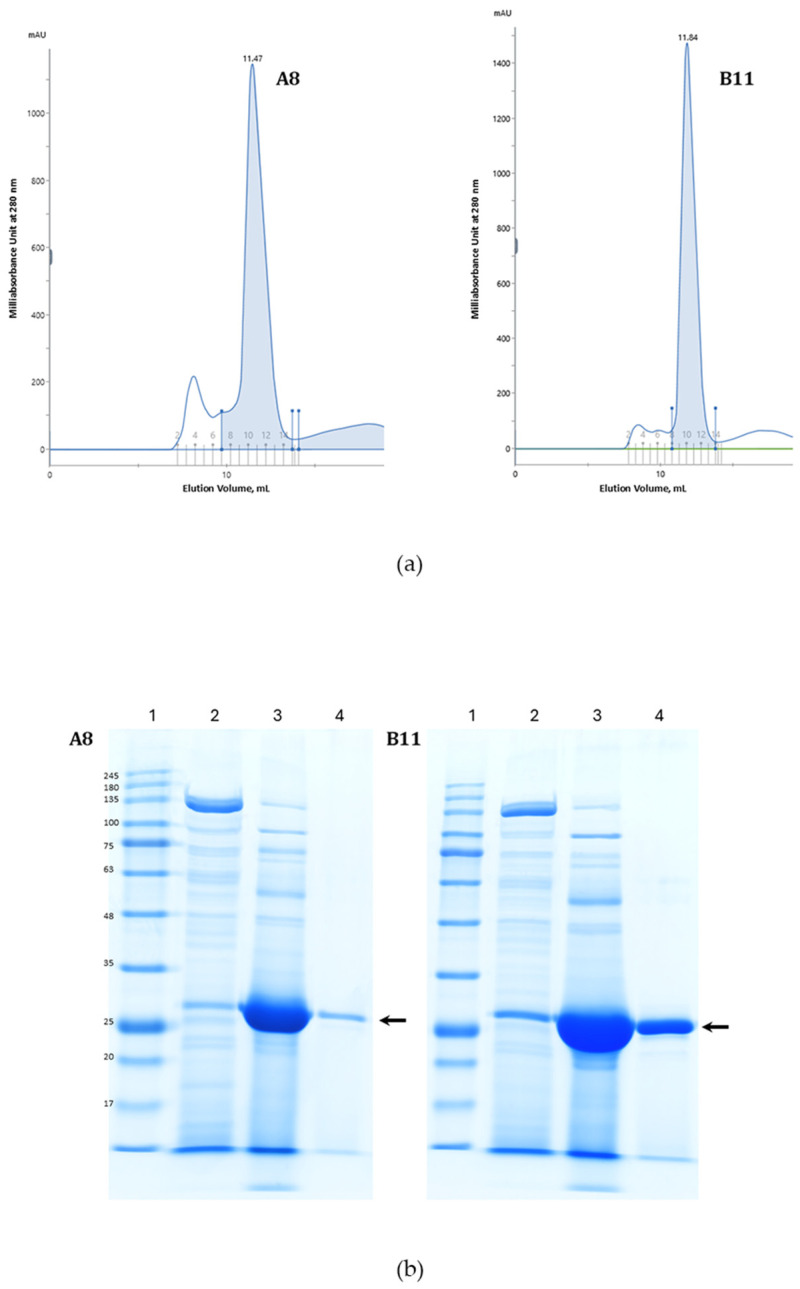
(**a**) Elution profile of ccombodies A8 and B11 in Superdex^TM^ 75 Increase 10/300 GL column. Shaded peaks correspond to the fractions pooled. (**b**) SDS-PAGE profile of purified ccombodies. Lane 1—molecular weight markers, 2—*E. coli* BL21(DE3) culture supernatant, 3—affinity purified proteins using nickel-charged magnetic beads, and 4—gel filtration chromatography pooled ccombody fractions (arrow).

The candidates were then cloned and expressed by IPTG induction in *E. coli* BL21. The presence of a 6×His-tag facilitated the affinity purification of the ccombodies from the complex mixture of bacterial proteins. The ccombodies were ultimately purified by size exclusion chromatography (Figure 2a). Both ccombodies showed similar molecular weights of ~27 kDa on SDS-PAGE (Figure 2b).

### 3.3. Ccombody Characterization

#### 3.3.1. Ccombody Specificity via ELISA

**Figure 3:**
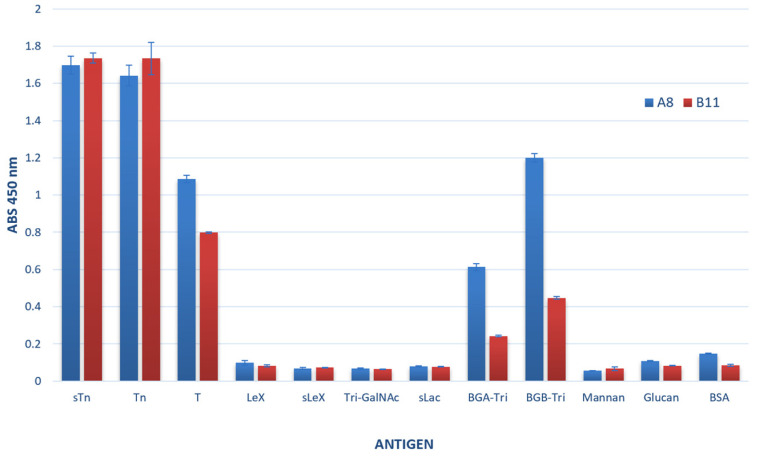
Elucidation of glycan motif bound by ccombodies A8 and B11. Both ccombodies exhibited preferential binding to glycans having monovalent α-GalNAc as the core sugar. Where sTn—sialyl Thomsen-nouveau; Tn—Thomsen nouveau; T—Thomsen–Friedenreich; LeX—Lewis X; sLeX—sialyl Lewis X; Tri-GalNAc—tri-antennary β-GalNAc; sLac—sialyllactosamine; BGA-Tri—Blood group A trisaccharide; BGB-Tri—Blood group B trisaccharide; Mannan—*Saccharomyces cerevisiae* D-Mannan; Glucan—*Saccharomyces cerevisiae* β-Glucan; BSA—bovine serum albumin (negative control). Values represent the mean of three measurements.

To evaluate the fine specificity of the ccombody candidates, we performed ELISA against a panel of structurally related and unrelated glycans (Figure 3). The results showed that both ccombodies preferentially recognized glycans containing α-GalNAc as the core sugar in an *O*-glycan context. However, binding affinity was also influenced by the distinct sugar moieties at the non-reducing end, as well as their respective glycosidic linkages. For instance, the strong binding to sTn and moderate binding to the T antigen suggests that the ccombodies can recognize α2-6 sialylation and β1-3 linked galactose extensions on the core α-GalNAc, respectively. Conversely, the ccombodies do not bind to glycans in which sialic acid is linked to an underlying sugar other than GalNAc, or when GalNAc is presented in a multivalent format, such as in tri-antennary β-GalNAc structures. Furthermore, both ccombodies recognized blood group A and B trisaccharides, with A8 exhibiting higher binding affinity than B11. These results indicate that terminal α-Gal is tolerated, although fucosylation may negatively impact binding.

#### 3.3.2. VVL and Ccombody Competition Assay

**Figure 4:**
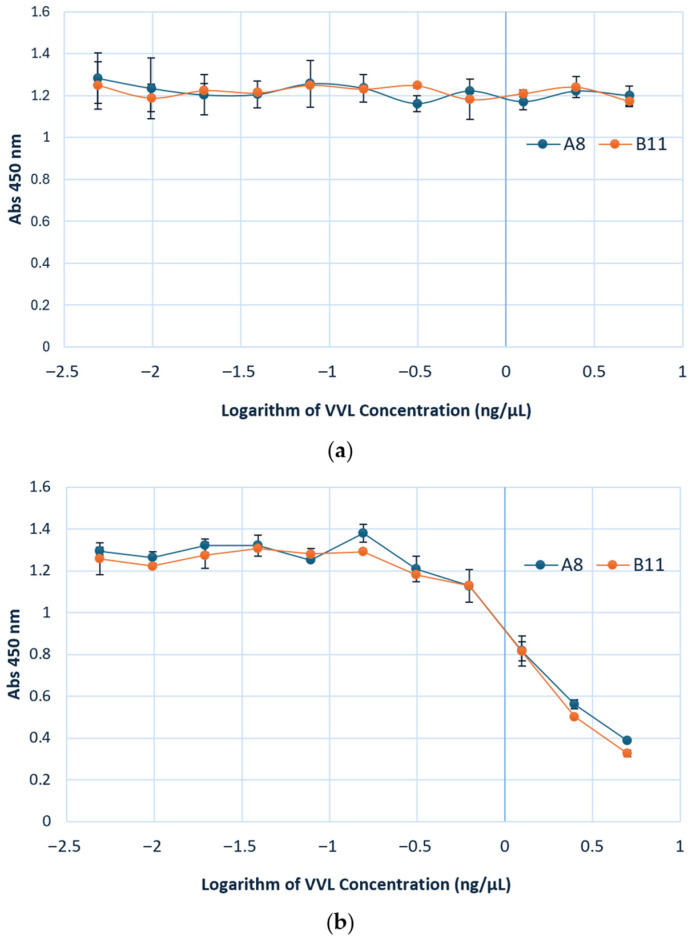
Lectin competition assay to assess the effect of increasing VVL concentration on the binding of ccombodies A8 and B11 to Tn and sTn antigens. (**a**) Tn antigen; (**b**) sTn antigen. The amount of VVL varied via a two-fold serial dilution from 5 to 0 ng/µL. Values represent the mean of two measurements.

While the specificity of VVL is well-established, exhibiting preferential binding to terminal α-linked GalNAc residues, we performed ELISA titration using VVL against the sTn antigen to confirm its ability to recognize this extended GalNAc-containing structure (Figure S1). After validating its binding to the sTn epitope, VVL was subsequently used in competition assays with the ccombodies to determine whether they target the same or overlapping glycan epitopes. The results of the competition assay reveal a dose dependent inhibition of ccombody binding to the sTn antigen with VVL as the competitor. The extent of inhibition was similar for both A8 and B11. At 5 ng/µL of VVL, the ccombody activities against sTn were 69% and 74% inhibited for A8 and B11, respectively (Figure 4b). These findings confirm that both ccombodies and VVL compete for overlapping epitopes, with reduction in ccombody binding at higher lectin concentrations most likely due to epitope masking or steric interferences brought about by VVL occupancy. Interestingly, the binding of both ccombodies to the Tn antigen was not substantially inhibited by VVL, suggesting that the lectin and ccombodies may recognize and interact with distinct, non-competing epitopes or structural planes of the molecule (Figure 4a).

#### 3.3.3. Ccombody Relative Affinity Expressed as EC_50_

**Figure 5:**
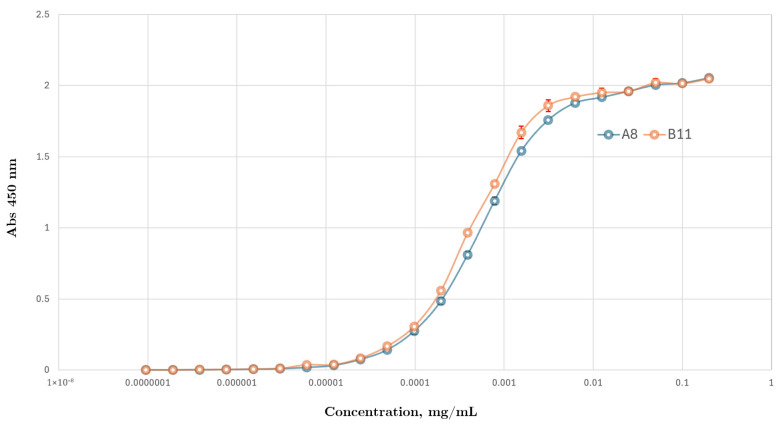
Dose–response curves of ccombodies A8 and B11 against sTn antigen. The inflection points for both curves are taken as the EC_50_ (in mg/mL) and approximate the relative binding affinity of the ccombodies toward the antigen. Values represent the mean of two measurements.

The magnitude of EC_50_ reflects the binding potency of both ccombodies against the sTn antigen, and further correlates with relative binding affinity under assay-specific conditions. Both ccombodies showed comparable EC_50_ values in the low nanomolar range, with values of 14.2 and 18.2 nM for B11 and A8, respectively (Figure 5).

#### 3.3.4. Binding with STn Expressed on Cells and Glycoproteins

**Figure 6:**
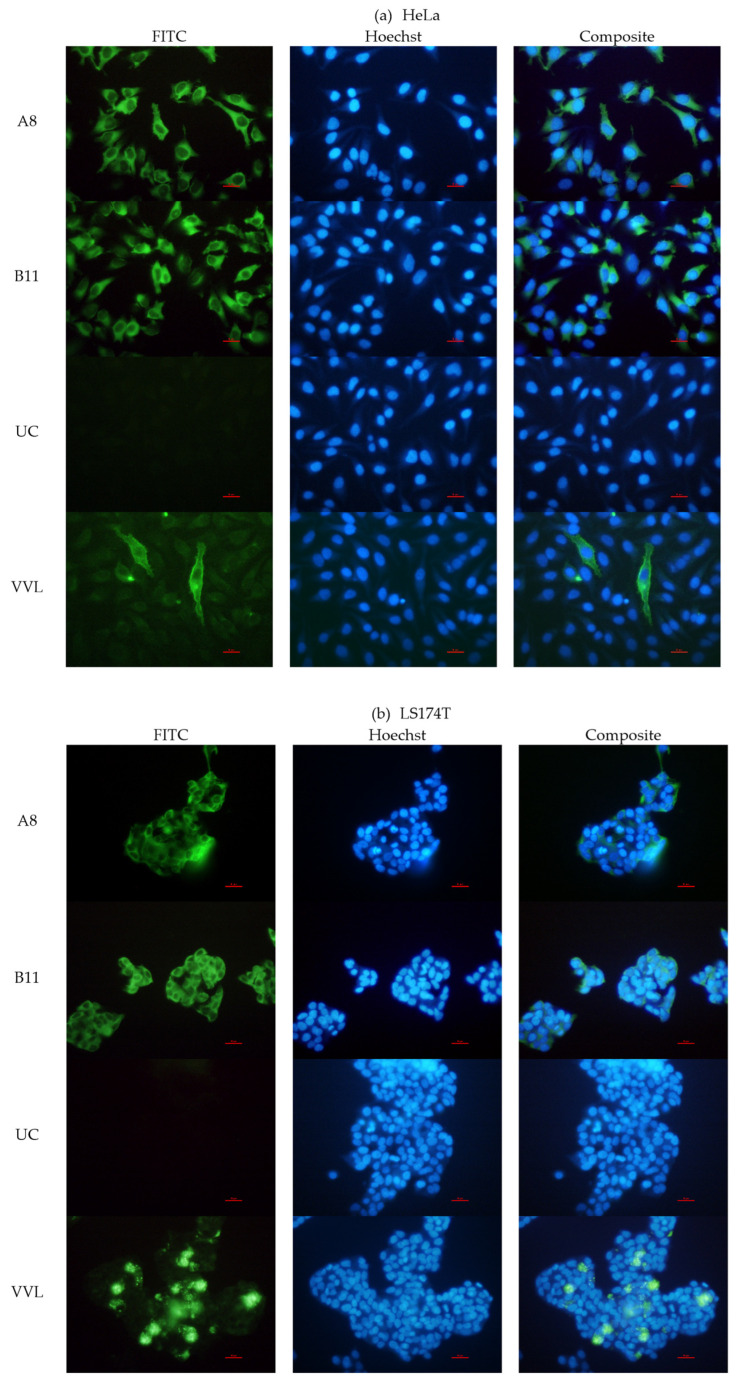

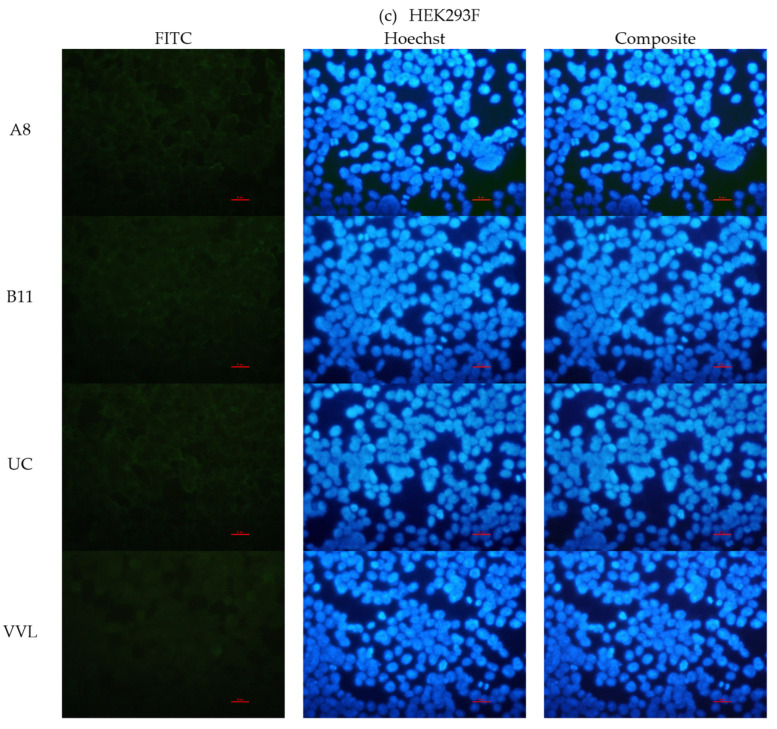
FITC-stained and composite fluorescence images (400× magnification) of carcinoma-derived cell lines (**a**) HeLa and (**b**) LS174T expressing the sTn antigen as detected by ccombodies A8 and B11. Cell nuclei were counterstained with Hoechst for reference. (**c**) HEK293F cell line and an unrelated ccombody (UC) were used as negative controls, while VVL served as a positive control. The red scale bar represents 10 µm.

We next evaluated the ability of the ccombodies to recognize naturally occurring sTn antigens, including structurally related glycans containing the monovalent α-GalNAc core. Carcinoma cell lines HeLa and LS174T were selected because they are known to express sTn antigens on their cell surfaces, as well as intracellularly by glycoproteins bearing aberrantly expressed glycans [11,30,31]. HeLa and LS174T are well-established human cell lines, derived from cervical carcinoma [32] and colon adenocarcinoma [33], respectively. In both cell lines, surface sTn antigens were accessible to glycan-binding proteins, as demonstrated by lectin (VVL) staining (Figure 6a,b). Immunofluorescence analysis further revealed distinct signals in both cell lines, indicating the probable binding of ccombodies A8 and B11 to their glycan targets (Figure 6a,b). In contrast, HEK293F, which is derived from transformed human embryonic kidney cells [34], showed no detectable fluorescence, consistent with the absence of tumor-associated antigens (Figure 6c).

**Figure 7:**
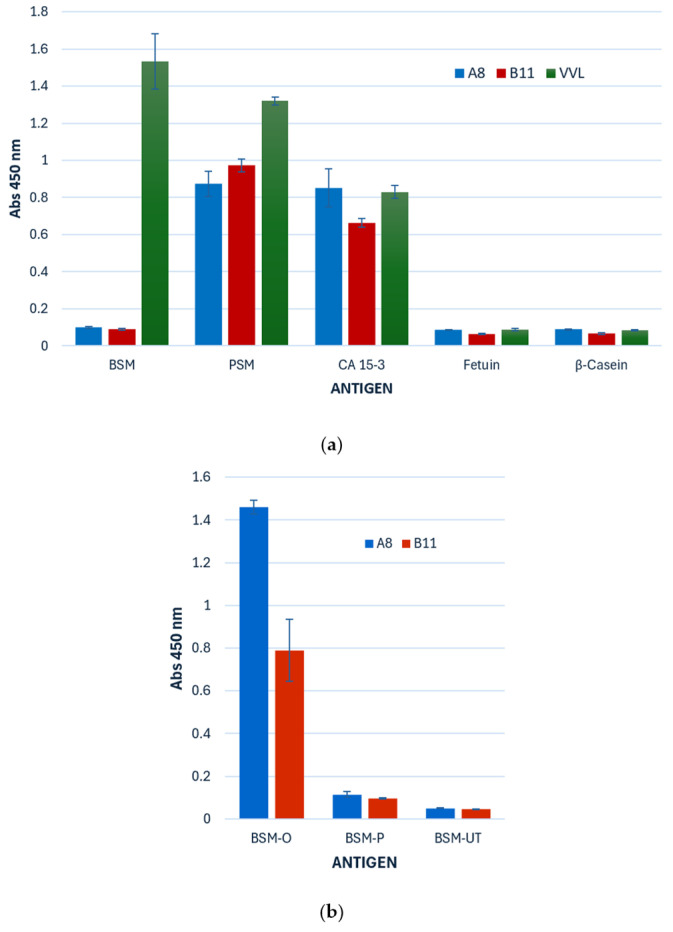
(**a**) ELISA binding profiles of anti-sTn ccombodies A8 and B11 against various glycoproteins. Where BSM—bovine submaxillary mucin; PSM—porcine stomach mucin; CA 15-3—cancer antigen 15-3; Fetuin—bovine fetuin; β-Casein—bovine milk β-casein (negative control); VVL—*Vicia villosa* lectin (positive control). (**b**) Binding of A8 and B11 to enzyme-treated BSM. Where BSM-O—*O*-glycosidase treated; BSM-P—PNGase treated; BSM-UT—untreated. Values represent the mean of two measurements.

Furthermore, ccombodies A8 and B11 exhibited binding to glycoproteins known to express the sTn antigen, particularly the highly glycosylated mucins (Figure 7a). As a reference, all three mucin samples were confirmed to contain the core α-GalNAc moiety, based on their reactivity with VVL. Both ccombodies recognized and bound CA 15-3, a mucin cancer antigen enriched with sTn and Tn structures. A8 and B11 also bound porcine stomach mucin (PSM), but neither exhibited binding to bovine submaxillary mucin (BSM) or bovine fetuin. Interestingly, both ccombodies only exhibited binding to BSM after *O*-glycosidase treatment, suggesting that the epitope may be masked or sterically hindered in its fully glycosylated form (Figure 7b). No ccombody binding was observed for PNGase-treated BSM.

## 4. Discussion

One of the most effective strategies for raising glycan-specific antibodies is through immunization using the desired glycan, which is often conjugated to a larger carrier molecule [35,36,37]. This approach compensates for the inherently poor immunogenicity of mammalian glycans due to their similarities with endogenous glycans, innate conformational flexibility, and limited capacity to stimulate T-cell responses [38,39,40]. Alternatively, numerous antibodies and other glycan-binding molecules targeting specific glycan antigens have been identified using high-throughput methods such as yeast surface and phage-display [39,41,42,43,44]. Synthetic VLR-based display systems have the advantage of leveraging libraries with repertoire designed to match or even exceed that of conventional antibodies. Moreover, the monomeric nature of the VLRB protein simplifies subsequent post-expression molecular techniques, including efficient amplification of the parent gene [15].

Like conventional antibodies, VLRBs are capable of binding diverse molecules. The modular nature of its LRR motifs imparts flexibility to the VLRB’s structure, adapting its size to complement that of the antigen’s epitope, thereby producing high affinity binders. In the context of glycan-binding, the LRRCT segment has a crucial role in securing the glycan within a crevice flanked on both sides by the LRRCT loop and LRR concave binding face. Therefore, amino acid residues near the C-terminus, particularly those that promote electrostatic-based interactions, such as H-bonding, ionic, and CH-π, are likely to be highly favored within the hypervariable regions [45,46,47]. These residues are expected to play key roles in both stabilizing the binding interface and mediating specific interactions with the glycan determinants. In ccombody A8, the non-canonical enrichment of aliphatic amino acids may confer the necessary van der Waals interactions that either enhance glycan selectivity or contribute to conformational stability of the binding site, similar to what has been reported for the glycan-specific VLRB.GPA.23 [17]. For example, TFα and H-trisaccharide-binding VLRs engage only ridges R2 and R3, which correspond to residues at positions 3 and 5–6 of the LRR hydrophobic core, respectively, when interacting with their glycan epitopes. In contrast, residues at ridge R1 do not directly participate in glycan binding but nonetheless contribute to maintaining the structural integrity of the LRR framework [17,48]. Furthermore, hydrophobic interactions are also key determinants of binding between sialic acid and many lectins [49]. Therefore, in our phage display screening for sTn-binding VLRBs, we introduced variability by randomizing the modules adjacent to the LRRCT loop to reflect the structural features observed in previously characterized glycan-binding VLRBs. The remaining unrandomized LRR modules can then be potentially targeted for further diversification, using in vitro directed evolution, to enhance both affinity and selectivity of the sTn-binding ccombodies. For instance, error-prone PCR combined with yeast surface display was utilized to introduce mutations in the VLRB diversity region, thus achieving in vitro affinity maturation, thereby yielding clones with superior affinities than the original [50].

The glycan-microenvironment has been found to significantly influence ccombody affinity and epitope recognition as demonstrated by other glycan-binding proteins like antibodies and lectins [12]. Several sTn-binding monoclonal antibodies have been shown to vary their affinities when the peptide context, epitope clustering, and steric shielding were altered [51,52,53,54]. Likewise, effective binding to the Tn epitope in MUC1 by VVL and soybean agglutinin has been shown to be dependent on the peptide sequence, with higher affinities observed when Tn is presented within the PDTR peptide segment [55]. For this reason, glycan array data obtained from VLRB screening should be cross-validated with native cell or tissue samples, as differences in scaffold architecture can significantly affect glycan presentation or accessibility [56]. In heavily glycosylated mucins, short glycans may be masked by extensive *O*-glycosylation, which creates a dense and heterogeneous outer glycan layer that prevents recognition by antibodies or lectins. However, deglycosylation, whether by chemical or enzymatic treatment, could reduce the glycan crowding and steric hindrance, thereby restoring accessibility. Consistently, BSM, which contains αGalNAc in an *O*-glycan context along with minor levels of sTn [57], was bound by ccombodies A8 and B11 only after partial enzymatic deglycosylation, suggesting that ccombody binding is similarly subject to the same microenvironment effects.

On the other hand, ccombodies A8 and B11 have demonstrated promising affinity and specificity in discriminating glycan epitopes, as shown in preliminary binding assays (Figure 3, Figure 5, and Figure S2). Only a limited number of glycan-binding VLRBs have been discovered thus far [16,50,58,59,60,61,62,63], and even fewer have been shown to recognize tumor-associated carbohydrate antigens [24,48,63,64]. Notably, mucins that are involved in diverse physiological and pathological functions have been extensively studied as biomarkers during tumor development, as they often transform into prominent carriers of TACAs following alterations in their glycosylation profiles [65,66,67]. CA15-3, a soluble fragment of MUC1 typically used as a serum biomarker for breast cancer, displays substantial amounts of TACAs such as sialyl-Lewis antigens, sialyl-Tn, Tn, sialyl-T, and T [68,69,70,71]. In addition, several mucin types with aberrant glycosylation are also expressed by LS174T cells, a human colorectal adenocarcinoma cell line [72,73]. While establishing the exact binding specificity of the ccombodies remains a challenge, as it requires comprehensive glycan arrays with diverse scaffold formats, alongside validation using natively expressed glycan sources, the capacity of A8 and B11 to recognize sTn-bearing glycoproteins and cells underscores their potential for the development of diagnostic tools in both clinical and therapeutic applications. However, their weak cross-reactivity with blood-group antigens may pose an important diagnostic caveat during analysis, particularly when applied to heterogeneous biological samples, as structurally related glycan motifs present on normal erythrocytes and epithelial tissues could reduce specificity. Therefore, to elucidate the molecular basis of ccombody-sTn interactions and enable the rational engineering of ccombodies with improved specificity and affinity for diverse applications, further structural and kinetic studies are warranted. Such studies should include high-resolution techniques such as X-ray crystallography or NMR, as well as binding assays employing surface plasmon resonance, biolayer interferometry, or microscale thermophoresis for the determination of the true equilibrium dissociation constant (K_D_).

In summary, biopanning of phage-displayed ccombody libraries represents a viable platform for discovering glycan-binding receptors with high specificity and affinity for tumor-associated glycoepitopes. Apart from their conventional application in basic research, ccombodies A8 and B11 show potential as valuable additions to the expanding repertoire of glycan-targeting reagents available to glycobiologists. With further optimization and validation, these ccombodies could be harnessed for a wide range of future applications, including glycan analysis, structure elucidation, diagnostics, and therapeutics.

## Figures and Tables

**Table 1:** Randomization sites of the ccombody phage library corresponding to the ccombody hypervariable region. Amino acids in red are the possible randomization sites. Actual randomized positions are represented by amino acid X.

Ccombody Segment	Sequence
LRR1	QIIANN
LRRV1	YLALGG
LRRV2	YLILTG
LRRV3	ELVLVE
LRRV4	XLXLXX
LRRV5	XLXLXX
LRRVe	XLXLXX
CP	XIXLXX

**Table 2:** Classification of amino acid residues in the randomized sites of ccombody A8 and B11.

Classification	Frequency	
	A8	B11
Acidic	1	3
Basic	5	3
Aromatic	1	5
Polar, Uncharged	3	3
Non-polar, Aliphatic	6	2

## Data Availability

The data presented in this study can be made available upon request to the corresponding author. Data is not publicly available due to privacy reasons.

## Supplementary Materials

The following supporting information can be downloaded at: https://www.mdpi.com/article/10.3390/cimb47100802/s1.

## Abbreviations

The following abbreviations are used in this manuscript:

BSA	Bovine serum albumin
BSM	Bovine submaxillary mucin
COSMC	core1-specific-molecular-chaperone
ELISA	Enzyme-linked immunosorbent assay
FITC	Fluorescein isothiocyanate
FPLC	Fast protein liquid chromatography
IPTG	Isopropyl β-D-1-thiogalactopyranoside
LRR	Leucine-rich repeats
PCR	Polymerase chain reaction
PSM	Porcine stomach mucin
SDS-PAGE	Sodium dodecyl sulfate-polyacrylamide gel electrophoresis
TACA	Tumor-associated carbohydrate antigen
TLR	Toll-like receptor
VLR	Variable lymphocyte receptor
VVL	Vicia villosa lectin
