# Supplementary material for: Phage Display Reveals VLRB-Mediated Recognition of Minimal Tumor Glycan Antigen Sialyl-Tn
Source: Curr Issues Mol Biol. 2025 Sep 26;47(10):802. doi: 10.3390/cimb47100802 (PMC12562327; doi:10.3390/cimb47100802)
Supplement: Supplementary file 1 [file cimb-47-00802-s001.zip › cimb-3834887-supplementary.pdf]

Supplementary Materials

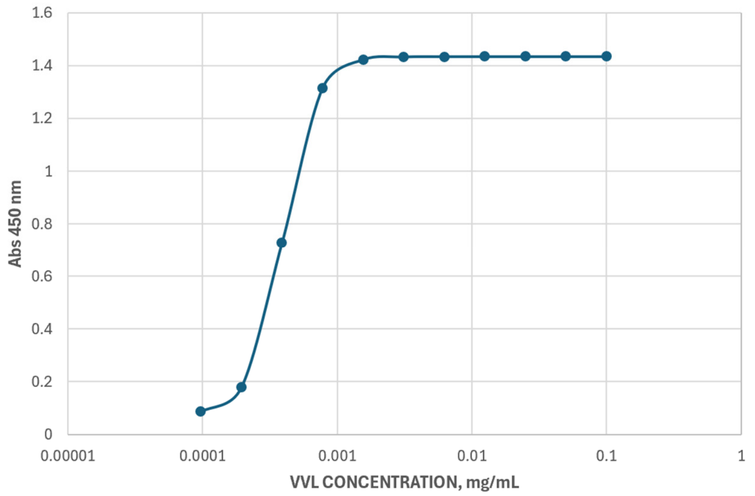

Figure S1. ELISA dose-response curve of VVL against the sTn antigen.

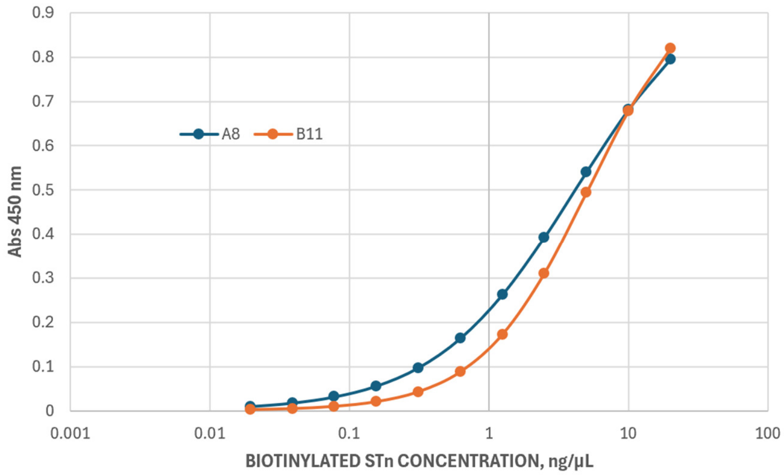

Figure S2. ELISA dose-response curve of ccombodies A8 and B11 against the sTn antigen. Sandwich ELISA was performed using the following conditions: ccombody coating concentration, 100 μg/mL; blocking agent, 1% BSA in TBST; biotinylated-sTn concentration, serial two-fold dilutions. (0 to 20 ng/μL); detection method, streptavidin-labeled peroxidase.
